# SMYD3 Controls Ciliogenesis by Regulating Distinct Centrosomal Proteins and Intraflagellar Transport Trafficking

**DOI:** 10.3390/ijms25116040

**Published:** 2024-05-30

**Authors:** Ewud Agborbesong, Julie Xia Zhou, Hongbing Zhang, Linda Xiaoyan Li, Peter C. Harris, James P. Calvet, Xiaogang Li

**Affiliations:** 1 Department of Internal Medicine, Mayo Clinic, Rochester, MN 55905, USA; 2 Department of Biochemistry and Molecular Biology, Mayo Clinic, Rochester, MN 55905, USA; 3 Department of Biochemistry and Molecular Biology, University of Kansas Medical Center, Kansas City, KS 66160, USA

**Keywords:** ciliogenesis, centriole, distal appendages, IFT trafficking, SMYD3

## Abstract

The primary cilium is a microtubule-based sensory organelle that plays a critical role in signaling pathways and cell cycle progression. Defects in the structure and/or function of the primary cilium result in developmental diseases collectively known as ciliopathies. However, the constituents and regulatory mechanisms of the primary cilium are not fully understood. In recent years, the activity of the epigenetic modifier SMYD3 has been shown to play a key role in the regulation of cell cycle progression. However, whether SMYD3, a histone/lysine methyltransferase, contributes to the regulation of ciliogenesis remains unknown. Here, we report that SMYD3 drives ciliogenesis via the direct and indirect regulation of cilia-associated components. We show that SMYD3 is a novel component of the distal appendage and is required for centriolar appendage assembly. The loss of SMYD3 decreased the percentage of ciliated cells and resulted in the formation of stumpy cilia. We demonstrated that SMYD3 modulated the recruitment of centrosome proteins (Cep164, Fbf1, Ninein, Ttbk2 and Cp110) and the trafficking of intraflagellar transport proteins (Ift54 and Ift140) important for cilia formation and maintenance, respectively. In addition, we showed that SMYD3 regulated the transcription of cilia genes and bound to the promoter regions of C2cd3, Cep164, Ttbk2, Dync2h1 and Cp110. This study provides insights into the role of SMYD3 in cilia biology and suggests that SMYD3-mediated cilia formation/function may be relevant for cilia-dependent signaling in ciliopathies.

## 1. Introduction

Primary cilia are microtubule-based organelles that protrude from the apical surface of most eukaryotic cells, where they function as signaling organelles, sensing and responding to extracellular mechanical and chemical cues [1]. In vertebrates, primary cilia have been associated with the regulation of embryonic development and tissue homeostasis [2]. As such, defects in cilia biogenesis and function result in a wide variety of human diseases collectively known as ciliopathies. The primary cilium originates from the basal body, which is derived from the mother centriole of the centrosome, anchored to the cell membrane [3]. The centrosome plays an essential function in nucleating the mitotic spindle during cell division. Prior to mitosis, the cell must resorb/disassemble the cilium to release the centrioles, and cilia assembly resumes shortly after cytokinesis. Thus, cell cycle progression is tightly associated with primary cilium assembly and disassembly. In recent years, studies have demonstrated that primary cilia play important roles in the development and progression of diverse hyperproliferative diseases. As such, an understanding of the regulation of primary cilia formation and function may provide insights into its mechanisms and reveal new therapeutic targets.

The primary cilium is divided into subdomains, including the ciliary pocket at the very base where the basal body is located [3] and the axoneme, the main body of the cilium, which emanates from the basal body into the cell surface [4,5]. The distal end of the basal body is the assembly site for two types of electron-dense projections called distal and subdistal appendages (DAs and SDAs, respectively) [2,6]. Proteins localized at these regions play essential roles in the regulation of ciliogenesis. Distal appendage proteins (DAPs) are key components of the microtubule organizing center (MTOC) and provide the foundation for primary cilium assembly. Defects or deficiencies in DAPs are associated with primary cilium malformation and/or function and alterations in the cilia-dependent signaling pathways and cause diverse developmental disorders in humans. Despite the importance of the distal appendage in ciliogenesis, its molecular composition and the mechanisms that guide its assembly and function are only beginning to emerge.

The distal appendage forms the transition fiber and serves as the docking site for vesicles carrying molecules for the cilia and connecting the cilium membrane and the basal body microtubules [4,5]. Distal to the transition fiber is the transition zone, a region of conversion from the microtubule triplet of the basal body to the microtubule doublet in the axoneme. The transition fibers, together with the transition zone, act as a “ciliary gate”, where the entry and exit of cilia proteins and lipids are regulated [4,6]. The transition fibers are also sites for the docking of intraflagellar transport (IFT) particles, which carry proteins and other cargo into the ciliary compartment. IFT is a process by which proteins are transported into and along the ciliary compartment. Anterograde IFT (IFT-B), in collaboration with the kinesin-2 motor, transports proteins along the cilium from base to tip, whereas retrograde IFT (IFT-A), together with the cytoplasmic dynein-2 motor, transports proteins from the tip to the base of the cilium [7]. Defects in the IFT system affect the trafficking and accumulation of cilia proteins, subsequently enhancing or inhibiting ciliogenesis.

SET- and MYND-domain-containing protein 3 (SMYD3), a histone/lysine methyltransferase, is a ubiquitously expressed protein belonging to a special class of protein lysine methyltransferases classified by the presence of the SET and MYND domains [8]. The SET domain is an evolutionarily conserved domain responsible for protein lysine methylation [9], while the MYND domain has a zinc finger motif involved in protein–protein interactions [10]. In many cell types, SMYD3 is located in the cytoplasm, with a fraction of the protein also located in the nucleus in several cancer cells [11]. SMYD3 has been reported to regulate the cell cycle progression of diverse types of human cancers. In these studies, it was found that the overexpression of SMYD3 played a significant role in enhancing the proliferation of cancer cells, whereas its downregulation inhibits cell proliferation. Moreover, SMYD3 promotes cell cycle progression by inducing cyclin D3 transcription, indicating that SMYD3 plays an intricate part in the regulation of the cell cycle [12]. The molecular mechanisms by which SMYD3 exerts its functions are largely limited to its roles in the epigenetic control of gene expression and in the recruitment of transcriptional cofactors [13]. In addition, SMYD3 also methylates non-histone proteins such as vascular endothelial growth factor receptor 1 (VEGFR1) to regulate VEGFR1 kinase activity, potentially affecting VEGFR1-dependent angiogenesis [14]. Based on its role in the regulation of cell cycle progression, SMYD3 might influence ciliogenesis by altering the transcription of cilia-related genes and/or target proteins via methylation.

Our study therefore aimed to investigate the role of SMYD3 in ciliogenesis and identify potential downstream cilia targets to elucidate the possible mechanism by which SMYD3 modulates ciliogenesis. We identified an unknown role of SMYD3 in the regulation of ciliogenesis and showed that SMYD3 is (1) a novel structural protein of the distal appendages of the mother centriole; (2) important for distal appendage assembly; (3) an adaptor for the recruitment of distal and subdistal appendage proteins; (4) a regulator of the trafficking of IFT proteins; and (5) an epigenetic regulator of ciliary gene transcription. This study identified SMYD3 as a novel regulator of ciliogenesis that may contribute to the pathogenesis of ciliopathies and advanced our understanding of epigenetic mechanisms in cilia biogenesis.

## 2. Results

### 2.1. SMYD3 Is Localized at the Basal Body and Regulates Cilia Biogenesis

**Figure 1:**
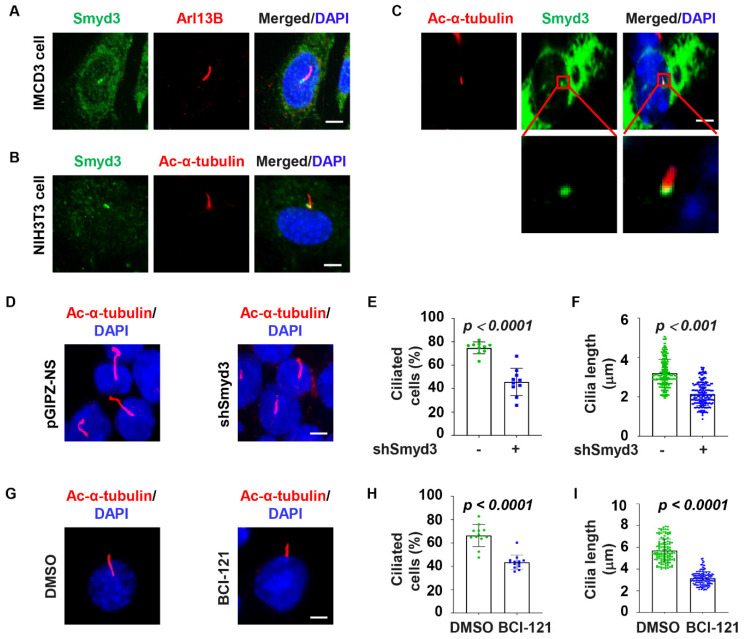
SMYD3 regulates ciliogenesis. (**A**) Representative images of IMCD3 cells stained with SMYD3 (green) antibody and co-stained with cilium marker, Arl13b (red), and counterstained with DAPI (blue). (**B**) Representative images of NIH3T3 cells stained with SMYD3 (green) antibody and co-stained with cilium marker, acetylated-α-tubulin (red), and counterstained with DAPI (blue). Scale bars: 5 μm. (**C**) Representative image of IMCD3 cells showing expression of exogenous GFP-SMYD3 (green), co-stained with acetylated-α-tubulin (red). The zoomed-in images are shown for better visualization. Scale bars: 5 μm. (**D**–**F**) Mouse IMCD3 cells were transduced with Lentivector-mediated SMYD3 shRNA, pGIPZ–shSMYD3, and the control vector, pGIPZ-NS, respectively. (**D**) Representative image, (**E**) quantification of the percentage of ciliated cells (*n* > 200), and (**F**) quantification of average cilia length (*n* > 200) in mouse IMCD3 cells transduced with shRNA control and SMYD3 shRNA and labeled with α-acetylated tubulin (red) and DAPI (blue). Scale bars: 5 μm. (**G**–**I**) Mouse IMCD3 cells treated with SMYD3 inhibitor (300 µM BCI-121) and the DMSO control. (**G**) Representative image, (**H**) quantification of the percentage of ciliated cells (*n* > 150), and (**I**) quantification of average cilia length (*n* > 175) in mouse IMCD3 cells treated with BCI-121 and labeled with α-acetylated tubulin (red) and DAPI (blue). Scale bars: 5 μm.

A growing body of evidence suggests that the regulators of the primary cilium are located at the centrosome and/or cilium axoneme. To investigate the role of SMYD3 in ciliary biogenesis, we first examined the subcellular localization of SMYD3 by immunostaining. We found that SMYD3 is localized at the basal body (mother centriole) of the cilia axoneme (marked with Arl13b) in mouse inner medullary collecting duct 3 (mIMCD3) cells (Figure 1A), a well-characterized cell line for ciliary studies. The localization of SMYD3 at the basal body was verified with a second SMYD3 antibody in mouse fibroblast NIH3T3 cells, co-stained with a second cilia axoneme marker, acetylated α-tubulin (Figure 1B). To further confirm the basal body localization of SMYD3, we expressed GFP-tagged SMYD3 (GFP-SMYD3) in mouse IMCD3 cells. Similar to endogenous SMYD3, we found that GFP-SMYD3 was also located at the base of the cilium (Figure 1C).

To investigate whether SMYD3 regulates ciliogenesis, we stably knocked down SMYD3 with shRNA in mouse IMCD3 cells. The efficiency of SMYD3 silencing was confirmed with qRT-PCR, Western blot, and immunostaining analyses in these cells (Supplemental Figure S1A,B). We found that the depletion of SMYD3 decreased the percentage of ciliated cells and the average cilia length compared to the control shRNA-transfected cells (Figure 1D–F). We further found that treatment with a SMYD3 inhibitor, BCI-121, decreased the percentage of ciliated cells and the cilia length in the mIMCD3 cells compared to those in the control cells treated with DMSO (Figure 1G–I). To further support the role of SMYD3 in the regulation of ciliogenesis, we examined whether the overexpression of GFP–SMYD3 affects cilia assembly in NIH3T3 cells, which had a higher GFP–SMYD3 plasmid transfection efficiency than the mIMCD3 cells. We found that the overexpression of GFP–SMYD3 increased the ciliated cell population and ciliary length in the NIH3T3 cells compared to those in the GFP-vector-transfected control cells (Supplemental Figure S1C–E). To determine whether the role of SMYD3 was conserved, we found that the knockdown of SMYD3 in human renal cortical tubular epithelial (RCTE) cells (Supplemental Figure S2A) decreased the percentage of ciliated cells and the average cilia length compared to the control siRNA-transfected cells (Supplemental Figure S2B–D). These results support a role for SMYD3 in the regulation of cilia biogenesis.

### 2.2. SMYD3 Is an Appendage Protein

**Figure 2:**
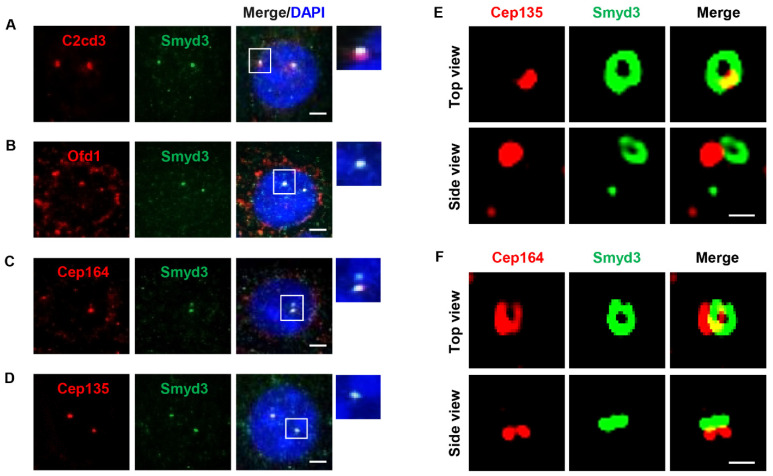
SMYD3 forms a ring-like pattern at the centrosome. (**A**–**D**) Two-dimensional immunofluorescence microscopy characterizing SMYD3 centrosome localization. Representative images indicate co-localization of SMYD3 (green) and centriole distal end markers (**A**) C2cd3 (red), (**B**) Ofd1 (red), the distal appendage marker (**C**) Cep164 (red) and the proximal end marker (**D**) Cep135 (red) in IMCD3 cells. (**E**) Three-dimensional SIM images of IMCD3 cells co-stained with SMYD3 (green) and Cep135 (red) antibodies indicated that SMYD3 forms a ring-like structure and Cep135 forms a dot beneath the ring. The top-view image shows that SMYD3 partially co-localizes with Cep135 in the centriole, and the side-view image shows that SMYD3 is located distal to Cep135 (bottom panels). Scale bars: 2 μm. (**F**) Three-dimensional SIM images of IMCD3 cells co-stained with SMYD3 (green) and Cep164 (red) antibodies indicated that both SMYD3 and Cep164 form a ring-like structure. The top-view image shows that SMYD3 partially co-localizes with Cep164, and the side-view image shows that SMYD3 is above Cep164. Scale bars: 2 μm.

To understand the mechanisms by which SMYD3 regulates ciliogenesis, we examined the precise localization of SMYD3 at the centriole. To do this, we co-stained SMYD3 with C2cd3 (C2 domain-containing protein 3) and Ofd1 (oral-facial-digital syndrome 1) (Figure 2A,B), markers for the distal region of the centriole; Cep164 (centrosomal protein 164) (Figure 2C), a distal appendage marker; and Cep135 (centrosomal protein 135) (Figure 2D), a marker for the proximal region of the centriole. Under 2D microscopy, SMYD3 co-localized with all these centrosome markers, so we were unable to determine the specific region of SMYD3 localization on the centriole. Interestingly, under 3D structured illumination microscopy (3D SIM), SMYD3 formed a ring-like structure, which was located distal to the centriolar wall protein Cep135 (Figure 2E) but partially overlapped with the distal appendage marker Cep164 (Figure 2F). These results suggest that SMYD3 is an appendage protein of the mother centriole and may play multiple roles between the mother and daughter centrioles.

### 2.3. SMYD3 Modulates Microtubule Stability

**Figure 3:**
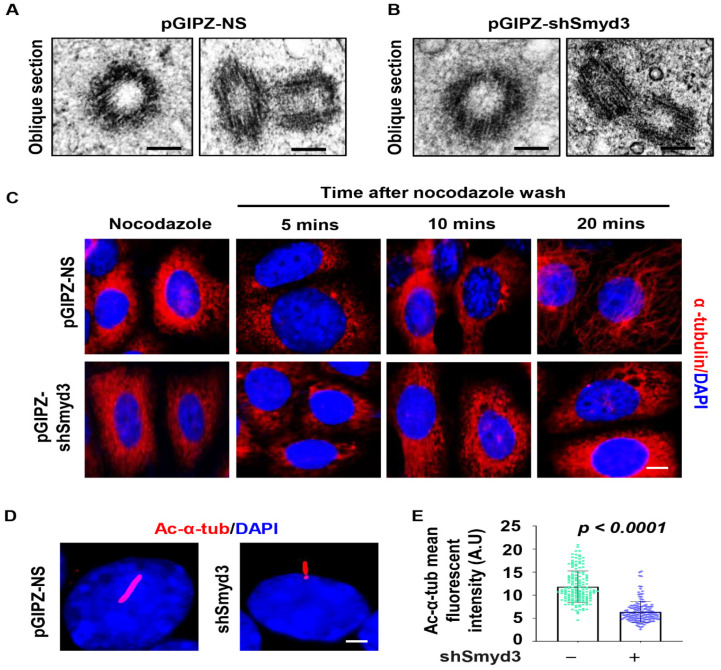
SMYD3 regulates microtubule regrowth. (**A**,**B**) Electron micrographs of the oblique sections of centrioles in control cells (**A**) and in SMYD3 knockdown IMCD3 cells (**B**). Scale bars: 500 nm. (**C**) Re-polymerization/regrowth of microtubules stained with α-tubulin (red) were delayed in SMYD3 knockdown mouse IMCD3 cells treated with nocodazole compared to control cells upon nocodazole release. After 5 min and 10 min, centriole and microtubule nucleation, respectively, were observed in control cells but were absent in SMYD3 knockdown cells. Scale bars: 5 μm. (**D**) Representative image and (**E**) mean fluorescent intensity measurement of the endogenous cilia marker acetylated α-tubulin (red) (*n* > 100).

We next wanted to determine the role SMYD3 played at the centrosome. Under transmission electron microscopy, we found that the microtubule triplet ultrastructure of the centrosome was not apparently disrupted in the SMYD3 stable knockdown IMCD3 cells compared to the control cells (Figure 3A,B). The centrosome is the major microtubule organizing center and serves as a nucleation site for microtubules in most cell types [15]. We next evaluated whether SMYD3 played a role in microtubule nucleation. To assess this, we performed a microtubule stability/regrowth assay. We treated mIMCD3 cells with nocodazole to depolymerize microtubules, then washed them with PBS and re-fed these cells with complete media on ice for microtubules to re-polymerize. We found that the re-polymerization of microtubules was delayed in the SMYD3-depleted cells compared to the control cells stained with α-tubulin (Figure 3C). Furthermore, we found that the depletion of SMYD3 decreased the fluorescence intensity of the ciliary marker acetylated-α-tubulin compared to the control mIMCD3 cells (Figure 3D,E). This suggests that the depletion of SMYD3 results in a decreased microtubule regrowth rate and/or stability, which may have contributed to the shortened cilia.

### 2.4. SMYD3 Regulates Centriolar Appendage Assembly

**Figure 4:**
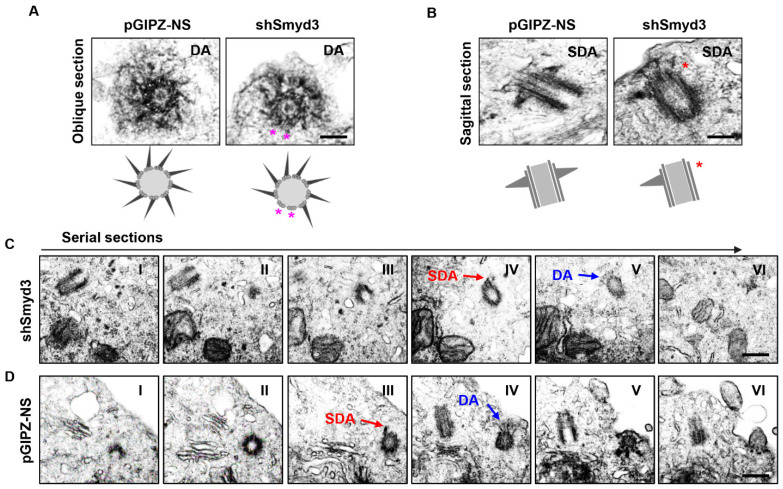
SMYD3 regulates appendage structure and centrosome docking. (**A**) Electron micrographs show the oblique sections (top panels) of the distal appendages, also illustrated in the diagrams (bottom panel). Pink stars indicate potentially missing distal appendage arms in SMYD3 knockdown cells compared to control cells. Scale bars: 500 nm. (**B**) Electron micrographs show the sagittal sections (top panels) of the subdistal appendages, also illustrated in the diagrams (bottom panel). Red stars indicate a potentially missing subdistal appendage arm in SMYD3 knockdown cells compared to control cells. Scale bars: 500 nm. (**C**,**D**) Representative electron micrographs showing serial sections of SMYD3-depleted (**C**) and control (**D**) IMCD3 cells serum-starved for 48 h. The red arrows indicate rootlets, and the purple arrows indicate distal appendages. Numbers I−VI represent consecutive serial sections. Scale bar: 500 nm.

**Figure 5:**
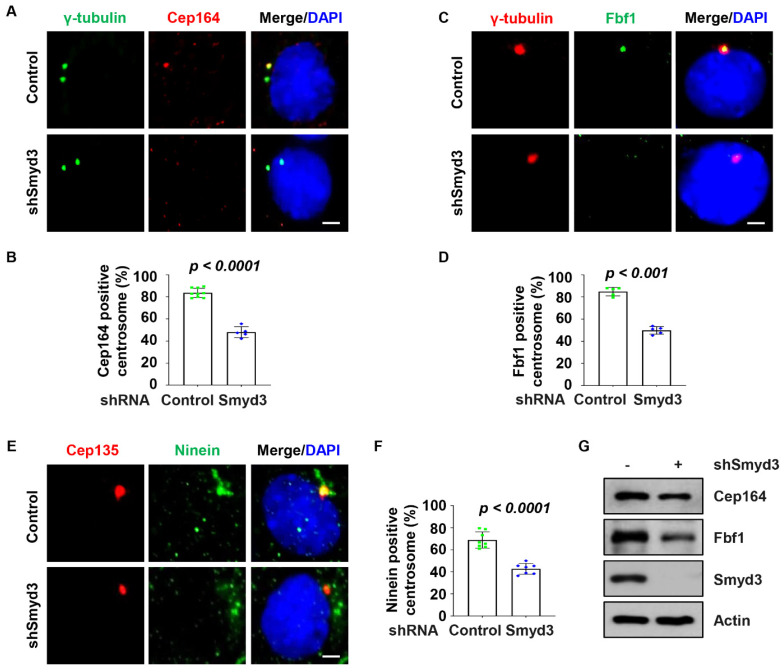
SMYD3 regulates the recruitment of centrosome appendage proteins. (**A**–**F**) Knockdown of SMYD3 decreased the recruitment of Cep164, Fbf1 and Ninein to the centrioles. (**A**) Representative image of Cep164 (red) co-stained with γ-tubulin and (**B**) quantitative analysis of the percentage of Cep164-positive centrioles (*n* > 275) in shRNA control or SMYD3-depleted IMCD3 cells. (**C**) Representative image of Fbf1 (green) co-stained with γ-tubulin and (**D**) quantitative analysis of the percentage of Fbf1-positive centrioles (*n* > 475) in shRNA control or SMYD3-depleted IMCD3 cells. (**E**) Representative image of Ninein (green) co-stained with Cep135 and (**F**) quantitative analysis of the percentage of Ninein-positive centrioles (*n* > 470) in shRNA control or SMYD3-depleted IMCD3 cells. (**G**) Western blot analysis of Cep164 and Fbf1 in SMYD3 shRNA knockdown IMCD3 cells.

To further investigate the role of SMYD3 at the centrosome, we further analyzed the centriole appendages by TEM. Oblique sections demonstrated that the centrioles of both the control and SMYD3-depleted cells were associated with distal appendages (Figure 4A), with the control cell centriole having nine arms, while the SMYD3-depleted cell centriole had seven, as depicted in the diagrams (Figure 4A, bottom panels). The sagittal sections also showed that the centrioles of both the control and SMYD3-depleted cells were associated with subdistal appendages (Figure 4B), with the control cell centriole having two arms, while the SMYD3-depleted cell centriole had one, as depicted in the diagrams (Figure 4B, bottom panels). By further analyzing serial sections of SMYD3 knockdown and control mouse IMCD3 cells, we observed that the rootlet was not fully formed in the SMYD3 knockdown IMCD3 cells (Figure 4C, section IV, red arrowhead) compared to that in the control cells (Figure 4D, section III). Furthermore, we observed thinner and shorter distal appendages in the SMYD3 knockdown IMCD3 cells (Figure 4C, section V, blue arrow) compared to the control cells (Figure 4D, section IV, blue arrow). Based on the orientation of the centrioles, we could not ascertain whether SMYD3 affected the centriole appendages. However, owing to the observations of the serial sections, we speculated that the depletion of SMYD3 might affect centrosome maturation/assembly. To assess this, we further found that the knockdown of SMYD3 disrupted the recruitment of the distal appendage proteins Cep164 (Figure 5A,B) and Fbf1 (Fas-binding factor 1) (Figure 5C,D) and the subdistal appendage protein Ninein (Figure 5E,F) to the basal body. The knockdown of SMYD3 also decreased the expression of Cep164 and Fbf1 in the mIMCD3 cells (Figure 5G). The downregulation of these proteins may be the cause of the decreased localization of Cep164 and Fbf1 to the basal body in the SMYD3-depleted cells. These results suggest that SMYD3 regulates the expression levels of the distal appendage proteins Cep164 and Fbf1, which subsequently influences their basal body recruitment and eventual regulation of cilia assembly.

### 2.5. SMYD3 Modulates TTBK2 and CP110 Basal Body Localization

Distal appendage assembly plays an essential role in cilia initiation. Cilia initiation requires the recruitment of the distal appendage protein TTBK2 (tau tubulin kinase 2) [16], which uncaps the mother centriole by removing CP110 (centrosomal protein 110), whose overexpression has been reported to suppress ciliogenesis [17]. To assess the role of SMYD3 in uncapping the mother centriole, we used RCTE cells since our TTBK2 antibody was reactive to human cells only. We found that the depletion of SMYD3 resulted in a decreased recruitment of TTBK2 to the basal body (Supplemental Figure S3A,B) and a decreased removal of CP110 from the basal body (Supplemental Figure S3C,D) compared to the control RCTE cells. The knockdown of SMYD3 also decreased the expression of TTBK2 and increased that of CP110 (Supplemental Figure S3E). Furthermore, we found that SMYD3 could form a complex with TTBK2, as examined by co-immunoprecipitation analysis (Supplemental Figure S3F), suggesting that TTBK2 may be a substrate for SMYD3. Our results suggest that the retention of CP110 at the basal body in SMYD3 knockdown cells may be a result of (1) the decreased recruitment of TTBK2 to the basal body and (2) the increased expression of CP110 in these cells.

### 2.6. SMYD3 Depletion Affects Ciliary Trafficking of IFT Proteins

**Figure 6:**
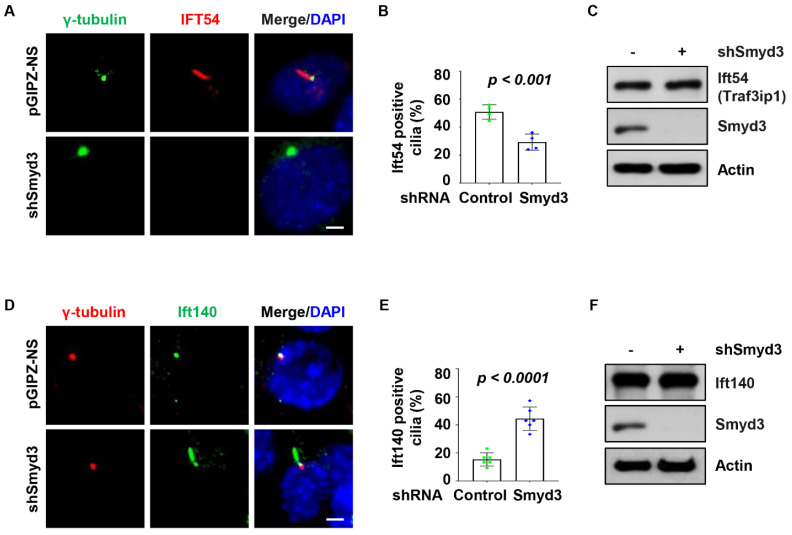
The depletion of SMYD3 regulates the trafficking of intraflagellar transport proteins. (**A**) Representative images and quantitative analysis of Ift54 cilia localization (**B**) in SMYD3 knockdown IMCD3 cells stained with Ift54 (red) and γ-tubulin (green). Knockdown of SMYD3 resulted in a decreased number of cells positive for Ift54. Scale bars: 5 μm. (**C**) Western blot analysis of Ift54 protein level in SMYD3 knockdown and shRNA control IMCD3 cells. Representative images (**D**) and quantitative analysis of Ift140 cilia localization (**E**) in SMYD3 knockdown IMCD3 cells stained with Ift140 (green) and γ-tubulin (red). Knockdown of SMYD3 resulted in an increased number of cells positive for Ift140. Scale bars: 5 μm. (**F**) Western blot analysis of Ift140 protein level in SMYD3 knockdown and shRNA control IMCD3 cells.

Intraflagellar transport, the bidirectional motility along axonemal microtubules, is essential for the formation and maintenance of cilia. We found that the knockdown of SMYD3 decreased the recruitment of Ift54 to the basal body and axoneme (Figure 6A,B) but did not affect the protein level (Figure 6C). In addition, the knockdown of SMYD3 increased the localization of Ift140 along the axoneme of the mIMCD3 cells (Figure 6D,E). Like Ift54, the knockdown of SMYD3 did not affect the protein level of Ift140 (Figure 6F). These results suggest that SMYD3 plays a vital role in regulating the dynamics of the IFT machinery by maintaining a balance between anterograde and retrograde movements. To further evaluate the role of SMYD3 in the regulation of IFT dynamics, we investigated whether SMYD3 is required for the ciliary trafficking of proteins involved in IFT regulation. We found that the knockdown of SMYD3 resulted in the cilia tip accumulation of the ciliary membrane-associated G-protein ARL13B (ADP-ribosylation factor-like protein 13B) (Supplemental Figure S4A–C), associated with retrograde IFT machinery [18], in the RCTE cells. We also found that the knockdown of SMYD3 increased the ciliary localization of BBS4 (Bardet–Biedl syndrome 4), a component of the BBSome complex associated with retrograde IFT proteins [19] (Supplemental Figure S4D–F) in RCTE cells. These results suggest that the depletion of SMYD3 may result in defective retrograde IFT machinery.

Retrograde IFT trafficking requires the dynein-2 microtubule motor complex, which includes the motor-domain-containing heavy chain (DYNC2H1), intermediate chains (WDR34), light intermediate chain (DYNC2LI1), light chain (DYNLT1), etc. [20,21,22,23]. Mutations in DYNC2H1 have been reported to result in the formation of cilia with bulbous tips [24,25]. We found the mRNA expression of DYNC2H1 to be significantly downregulated (Supplemental Figure S5A), while the mRNA expressions of DYNLT1 and WDR34 were significantly upregulated in the SMYD3-depleted RCTE cells (Supplemental Figure S5B). Our results suggest that the downregulation of DYNC2H1 may contribute to the defects in the retrograde transport of ciliary proteins in SMYD3-depleted cells.

### 2.7. SMYD3 Regulates the Transcription of Cilia-Related Genes

**Figure 7:**
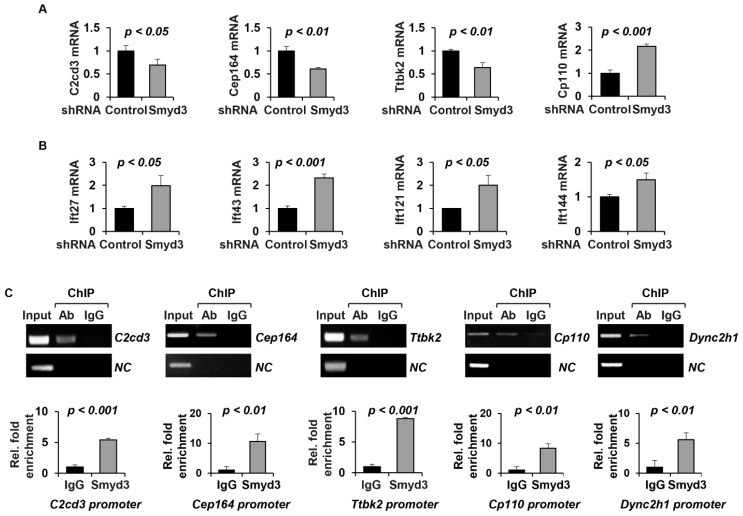
SMYD3 binds to the promoter region of cilia-related genes. (**A**) qRT-PCR analysis of mRNA levels of key components of the centrosome, including C2cd3, Cep164, Ttbk2, and Cp110, in SMYD3 knockdown IMCD3 cells compared to shRNA control IMCD3 cells. (**B**) qRT-PCR analysis of mRNA levels of components of the anterograde transport complex, including Ift27, and the retrograde transport complex, including Ift43, Ift121 and Ift144, in SMYD3 knockdown IMCD3 cells compared to shRNA control IMCD3 cells. (**C**) SMYD3 regulates the transcription of cilia-related genes by binding to their promoters. ChIP-qPCR analysis shows that SMYD3 binds to the promoter region of C2cd3, Cep164, Ttbk2, Cp110 and Dync2h1. ChIP and ChIP-qPCR assays were performed with anti-SMYD3 antibody and normal rabbit IgG (negative control) in mouse IMCD3 cells. NC represents negative control primers designed 3000 bp above transcription start site.

The knockdown of SMYD3 modulated the levels of cilia proteins. Since SMYD3 is known to regulate gene transcription [26], we next wanted to determine whether SMYD3 may play a role in the transcription of ciliary genes. We found that the knockdown of SMYD3 decreased the mRNA levels of the centrosomal genes C2cd3, Cep164 and Ttbk2 but increased that of Cp110 in the mIMCD3 cells (Figure 7A). In addition, the knockdown of SMYD3 increased the mRNA levels of the retrograde IFT genes Ift27, Ift43, Ift121 and Ift144 (Figure 5B) in the mIMCD3 cells. However, we found that the knockdown of SMYD3 did not affect the transcription of the centrosome genes Cc2d2a (coiled-coil and calcium binding domain protein 2A), Fbf1 and Ofd1; the anterograde IFT-related genes Ift52, Ift54 and Ift88; or the retrograde IFT-related genes Ift122 and Ift139 (Supplemental Figure S5C–E). This suggests that SMYD3 may be involved in the direct transcription of centrosome-associated genes. To investigate whether SMYD3 directly regulates the transcription of the above ciliary genes, we performed chromatin immunoprecipitation (ChIP) assays coupled with quantitative PCR (ChIP-qPCR) analysis with the SMYD3 antibody. We found that SMYD3 bound to the promoters of centrosomal genes, including C2cd3, Cep164, Ttbk2 and Cp110 (Figure 7C). In addition, we found that SMYD3 bound to the promoter of Dync2h1 (Figure 7C). These results suggest that SMYD3 plays a role in ciliogenesis via the epigenetic regulation of ciliary gene transcription.

## 3. Discussion

**Figure 8:**
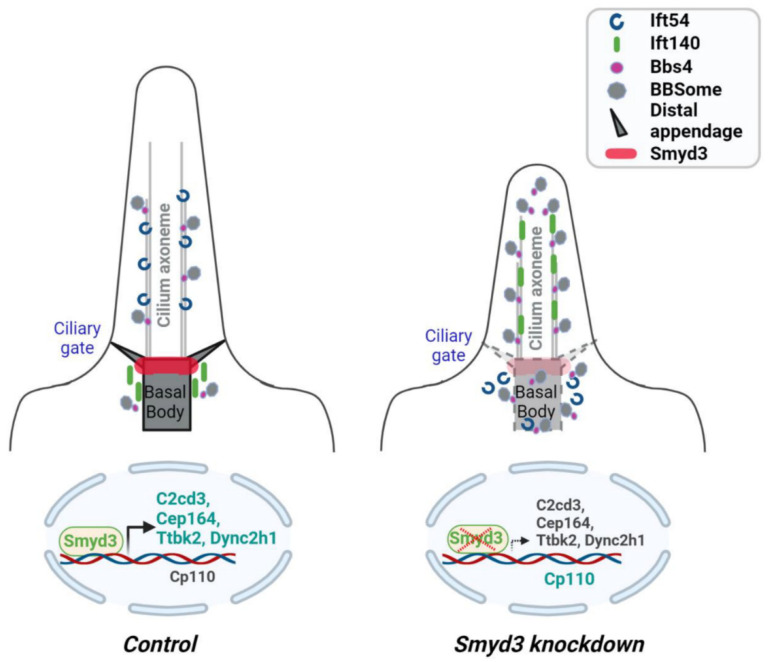
Working model of SMYD3 in the regulation of ciliogenesis. A schematic representation of SMYD3-mediated mechanisms in ciliogenesis. The knockdown of SMYD3 inhibits ciliogenesis. SMYD3 regulates ciliogenesis by functioning as (i) a structural protein at the basal body, which stabilizes the centrosome’s ultrastructure; (ii) an adaptor at the centrosome to regulate appendage assembly through the recruitment of Cep164, Fbf1, Ninein and Ttbk2 and the removal of Cp110 at the centrosome; (iii) a modulator of IFT trafficking and (iv) an epigenetic factor that regulates the transcription of ciliary genes, including C2cd3, Cep164, Ttbk2, Cp110 and Dync2h1, by binding to their promoters. The depletion of SMYD3 inhibits ciliogenesis by (a) decreasing the recruitment of Cep164, Fbf1 and Ttbk2 (required for cilia initiation) to the centrosome; (b) increasing the retention of Cp110 (inhibitor of cilia initiation) at the centrosome; (c) and decreasing the transcription of the C2cd3, Cep164, Ttbk2 and Dync2h1 genes, which are required for cilia initiation and retrograde IFT trafficking, respectively, while increasing the transcription of the Cp110 gene. Created with BioRender.com, accessed on 16 March 2024.

Deciphering the mechanisms that regulate ciliogenesis is of paramount importance since defects in primary cilia formation and/or function leads to developmental diseases. In this study, we reveal that SMYD3 is a novel component of the distal appendage and is important for proper centrosome assembly. We show that SMYD3 depletion impedes cilia initiation by decreasing the recruitment of the distal appendage proteins Cep164, Fbf1 and TTBK2 and the subdistal appendage protein Ninein to the basal body but increasing the retention of CP110 at the basal body. In addition, we show that SMYD3 plays a role in ciliogenesis by modulating the IFT machinery via Ift54 and Ift140 and via ARL13B and BBS4 ciliary trafficking. Finally, we show that SMYD3 plays a bi-faceted function as a transcriptional activator and a transcriptional repressor: (i) the knockdown of SMYD3 resulted in the downregulation of C2cd3, Cep164, Ttbk2 and Dync2h1 mRNA and the upregulation of Cp110 mRNA and (ii) SMYD3 bound to the promoter regions of C2cd3, Cep164, Ttbk2, Dync2h1 and Cp110 (Figure 8). This study demonstrates that the histone methyltransferase, SMYD3, plays distinct roles in the spatiotemporal organization of the primary cilium, thus suggesting that SMYD3 may play a role in the pathophysiology of ciliopathy-related diseases.

SMYD3 is a vital component of the RNA polymerase complex and primarily catalyzes H3K4me3. Its functions have been largely studied in cancer for tumorigenesis. A number of inhibitors targeting SMYD3 have been developed, but in this study, we only tested BCI121. We found that targeting SMYD3 with BCI121 inhibited cilia growth in cultured IMCD3 cells, similar to shRNA-depleted SMYD3 IMCD3 cells. In cancer studies, reports indicate that the treatment of cancer cells with a BCI-121 inhibitor results in cell arrest at the S phase of the cell cycle [27]. This may explain why treatment with BCI-121 inhibited cilia growth in the cultured IMCD3 cells since cilia growth is reported to be most prominent at the G0/G1 phase of the cell cycle. It is important to mention that BCI-121 is a substrate-competitive inhibitor of SMYD3, impairing histone methylation (H3K4 and H4K5) [28]. Therefore, one cannot rule out the possibility that its effect on ciliogenesis is indirect via histone methylation.

An important role in primary cilia biogenesis resides at distal appendages [4], which are critical for anchoring the basal body underneath the plasma membrane and for the early docking of Golgi-derived membrane vesicles. Distal appendages decorate the periphery of the distal end of the mother centriole and exhibit a ring-like pattern, as demonstrated by immunofluorescence analysis and super-resolution imaging [29]. Cep164 and Ttbk2 co-localize to distal appendages, and their interaction is well established. Cep164 is proposed to mediate cilia initiation by recruiting active Ttbk2 to the centrioles [30]. Once in place, Ttbk2 then triggers events required for ciliogenesis, including the removal of Cp110 and the recruitment of intraflagellar transport proteins. Furthermore, previous studies hint at the existence of a positive feedback loop centered on the Cep164–TTBK2 complex [30]. In this study, we demonstrated that SMYD3 may be a novel distal appendage component, supported by the observations that (1) SMYD3 forms a ring-shaped structure which associates with the distal appendage protein Cep164 and (2) SMYD3 forms a complex with the distal appendage protein TTBK2. In addition, we have demonstrated that SMYD3 plays a key role in the recruitment of Cep164 and Ttbk2 and the removal of Cp110 from the mother centriole. Our studies suggest that SMYD3 may interact with Ttbk2 to regulate its activation, thereby influencing downstream Ttbk2-targeted events. Further, the molecular characterization of the link between SMYD3 and Ttbk2 will be the subject of future studies in our lab.

SMYD3 is a histone lysine methyltransferase known to modulate the function of non-histone substrates via post-translational modifications. Whether SMYD3 plays any role in the cytoplasmic microtubule network and dynamics is unknown. Our microtubule regrowth assay shows that the microtubules grow slower in the SMYD3 knockdown cells than the control cells, although the microtubule network appears similar. This suggests that SMYD3 promotes cytoplasmic microtubule growth. Recent studies have shown that microtubule stability is regulated by SMYD2, a histone lysine methyltransferase of the same family as SMYD3, via the methylation of α-tubulin at lysine 40 [31]. Therefore, additional studies are necessary to elucidate the mechanism underlying this novel role of SMYD3 and whether there exists any redundancy between SMYD3 and SMYD2 in their lysine methylation sites of α-tubulin.

Anterograde (IFT-B) ciliary protein trafficking is powered by kinesin 2 motors, while retrograde (IFT-A) ciliary protein trafficking is powered by cytoplasmic dynein 2 motors. Disrupting the functions of the IFT cargo adaptor complexes (IFT-A and IFT-B) or the IFT motors leads to defects in cilia biogenesis [32]. The depletion of or mutations in the dynein 2 genes, for example, result in stumpy cilia and the aberrant accumulation of proteins such as Arl13b and Ift140 in the cilia and cilia tip [33,34]. We have demonstrated that the knockdown of SMYD3 increased the ciliary presence of Ift140 and BBS4 and enriched Arl13b at the cilia tips. Moreover, we found that the knockdown of SMYD3 resulted in the downregulation of the dynein heavy chain gene (Dync2h1). Consistent with the specific role of dynein 2 motors in retrograde IFT trafficking, our data suggests that SMYD3, via Dync2h1, is necessary for retrograde trafficking.

Reports have indicated that the enrichment of Arl13b and BBS4 plays a role in regulating the dynamic ciliary movement of Hedgehog signaling components and the sensory receptor polycystin 2 [35]. The role of primary cilia in hedgehog signaling is complex and context-dependent, and cilia can function both as positive and negative mediators of the hedgehog signaling pathway. In this study, we found that the knockdown of SMYD3 resulted in the enrichment of ARL13B and BBS4 along the cilia axoneme. Our results suggest that SMYD3 may play a role in the activation or repression of the hedgehog signaling pathway. Our results also suggest that SMYD3 may play a role in regulating the ciliary localization of polycystin 2. Mutations in polycystin 2 are known to cause ADPKD (autosomal dominant polycystic kidney disease) [36], suggesting that SMYD3 may contribute to cyst growth in ADPKD in a cilia-dependent manner.

The maintenance of cilia is also likely to require the continuous transcription and translation of ciliary components. SMYD3 is a histone methyltransferase that regulates gene transcription through the methylation of the histone H3K4 and/or directly binding to the regulatory regions of target genes [37,38]. We found that the knockdown of SMYD3 resulted in the downregulation of C2cd3, Cep164, Ttbk2 and Dync2h1. Interestingly, the knockdown of SMYD3 also resulted in the upregulation of Cp110, Ift27, Ift43, Ift121 and Ift144. Our ChIP assays of SMYD3 on ciliary genes, including C2cd3, Cep164, Cp110, Ttbk2 and Dync2h1, confirmed that SMYD3 might regulate the transcription of these genes via direct binding to their promoters. These results suggest that the SMYD3-mediated transcriptional regulation of ciliary genes is one of the mechanisms that regulate cilia formation and cilia length.

During the cell cycle, the CP110 level is tightly controlled by proteasome-mediated degradation. At least two ubiquitin ligases have been linked to CP110 degradation. The SCF (Skp1–Cul1–F-box) protein ubiquitin ligase and its substrate recognition subunit, the F-box protein cyclin F, bind to CP110 and control its ubiquitylation and degradation [39]. However, the transcriptional regulation of Cp110 has not been reported. In this study, we showed that the knockdown of SMYD3 modulated the transcription of Cp110, as demonstrated by the increased mRNA level. Furthermore, we found that SMYD3 binds to the promoter region of the Cp110 gene. SMYD3 is mostly known for its role in the activation of gene transcription. However, our data indicate that SMYD3 represses Cp110 gene transcription. This raises the possibility of an intermediate binding partner rather than the direct binding of SMYD3 to the promoter of Cp110. Further studies are necessary to investigate this possibility.

In summary, we have identified, for the first time, that the epigenetic regulator SMYD3 is a structural component of the centrosome/basal body. In addition to its role as a transcriptional regulator for the specific ciliary genes shown in this study, SMYD3 (i) facilitates the recruitment of distal appendage proteins; (ii) forms a complex with TTBK2; and (iii) regulates the trafficking of IFT proteins, ARL13B and BBS4. Understanding the functional consequences of the interaction between SMYD3 and TTBK2 and SMYD3’s interactions with other components implicated in ciliogenesis will undoubtedly be critical for a mechanistic understanding of ciliogenesis and may shed light on a previously unknown role for SMYD3 in the pathophysiology of ciliopathies.

## 4. Materials and Methods

### 4.1. Cell Culture

Mouse inner medullar collecting duct (mIMCD3) [ATCC, Cat# CRL-2123], NIH3T3 [ATCC, Cat# CRL-1658], and HEK293T [ATCC, Cat# CRL-3216] cells were maintained at 37 °C in 5% CO_2_ in DMEM (Invitrogen, Waltham, MA, USA) supplemented with 10% FBS. RCTE [ATCC, Cat# PCS-400-010] cells were maintained in DMEM (Invitrogen) supplemented with 10% FBS at 37 °C in 5% CO_2_. All cells were purchased from American Type Culture Collection (ATTC). For ciliary assembly analysis, cells were plated at 70% confluency in plates containing glass coverslips, grown until confluent, and starved for 24 h–48 h (regular DMEM without serum) to induce cilia formation, followed by immunostaining.

### 4.2. Plasmids

The GFP-tagged SMYD3 plasmid was constructed by cloning full-length SMYD3 into the pAcGFP-C1 vector (Clontech Laboratories, Inc. Mountain View, CA, USA) and pCMV-C-Flag vector.

### 4.3. Generating Stable SMYD3 Knockdown IMCD3 Cell Line

HEK293T cells were co-transfected with the lentiviral plasmid pGIPZ–shSMYD3 carrying SMYD3 shRNA or a control empty vector, pGIPZ-NS, the psPAX2 packaging plasmid and the pMD2.G envelope plasmid using calcium phosphate. Twelve hours later, the medium containing the transfection reagent was removed and replaced with fresh complete DMEM with 10% FBS and penicillin/streptomycin. Forty-eight hours later, cultures containing lentiviral particles were harvested from HEK293T cells. IMCD3 cells were then infected with appropriate amounts of lentiviral particles together with 5 µg/mL of polybrene (Sigma, St Louis, MO, USA). Twenty-four hours later, the virus-containing medium was removed and replaced with fresh medium plus 10 µg/mL of puromycin. Two days after selection, all the cells were GFP-positive, which indicated the remarkably high efficiency of transduction. Subsequently, a single clone of cells was chosen and cultured in the medium with 10 µg/mL of puromycin for Western blot and immunostaining analyses.

### 4.4. Drug Treatment

mIMCD3 cells were treated with nocodazole (1 µg/mL, Sigma-Aldrich, St. Louis, MO, USA) and BCI-121 (300 µM, Sigma-Aldrich).

### 4.5. Antibodies and Reagents

The primary antibodies used in this study included the following: mouse monoclonal antibodies against SMYD3 (Sigma, SAB4200344, 1:300 used for immunofluorescence (IF) and 1:1000 for Western blot (WB)), acetylated α-tubulin (6-11B-1, Sigma, T7451, 1:4000 used for IF), γ-tubulin (GTU-88, Sigma, T5326, 1:1000 used for IF), α-tubulin (DM1A, sc-32293, 1:2000 used for IF), Ninein (F-5, Santa Cruz, sc-376420, 1:500 for IF) and actin (AC-15, Sigma, A1978, 1:4000); rabbit polyclonal antibodies against SMYD3 (ThermoFisher, Waltham, MA, USA, PA5-31919, 1:300 used for IF and 1:1000 for WB), SMYD3 (Proteintech, Rosemont, IL, USA, 12011-1-AP, used for ChIP), C2cd3 (ThermoFisher, PA5-72860, 1:300 used for IF), Cep135 (Abcam, San Diego, CA, USA, ab75005, 1:300 used for IF), Cep164 (Proteintech, 2227-1-AP, 1:300 used for IF and 1:1000 for WB), Ofd1 (Abcam, ab222837, 1:300 used for IF), Fbf1 (Proteintech, 11531-1-AP, 1:300 used for IF and 1:1000 for WB), TTBK2 (Sigma, HPA018113, 1:300 used for IF and 1:1000 for WB), Cp110 (Proteintech, 12780-1-AP, 1:300 used for IF and 1:1000 for WB), IFT88 (Proteintech, 13967-1-AP, 1:300 used for IF), IFT140 (Proteintech, 17460-1-AP, 1:300 used for IF and 1:1000 for WB), ARL13B (Proteintech, 17711-1-AP, 1:2000 for IF) and BBS4 (Proteintech, 12766-1-AP, 1:300 used for IF); and TRAF3IP1/IFT54 (Proteintech, 14404-1-AP, 1:300 used for IF and 1:1000 for WB) and Pericentrin (EMD Millipore, Burlington, MA, USA, ABT59, 1:1000 used for IF). All secondary antibodies for immunofluorescence (goat anti-mouse; rabbit or donkey anti-mouse; rabbit or goat conjugated with Alex Fluor 488, 555, or 647) were purchased from Invitrogen, and the secondary antibodies for the Western blot, including donkey anti-rabbit IgG–horseradish peroxidase (sc-2313), donkey anti-goat IgG–horseradish peroxidase (sc-2020) and goat anti-mouse IgG–horseradish peroxidase (sc-2005), were purchased from Santa Cruz Biotechnology Inc., Dallas, TX, USA.

### 4.6. Immunofluorescence Staining

Cells grown on coverslips were rinsed with 1x phosphate-buffered saline (PBS) and fixed in cold methanol for 10 min at −20 °C, followed by permeabilization with 0.2% Triton X-100 for 15 min at 37 °C, or cells were fixed with 4% paraformaldehyde (PFA) for 10 min at 37 °C, followed by permeabilization with 0.2% Triton X-100 at room temperature for 10 min. Cells were then washed by PBS three times, blocked in 2% BSA and sequentially incubated with primary and secondary antibodies.

### 4.7. Western Blot Analysis and Immunoprecipitation

Cell pellets were collected and re-suspended in lysis buffer (20 mM Tris-HCl, pH 7.4, 150 mM NaCl, 10% glycerol, 1% Triton X-100, 1 mM Na_3_VO_4_, 25 mM β-glycerol-phosphate, 0.1 mM PMSF, Roche complete protease inhibitor set, and Sigma-Aldrich phosphatase inhibitor set). The re-suspended cell pellet was vortexed for 20 s and then incubated on ice for 30 min and centrifuged at 20,000× *g* for 30 min. The supernatants were collected for Western blot analysis or immunoprecipitation.

For immunoprecipitation, anti-SMYD3 and anti-TTBK2 antibodies and their isotype control antibodies were coupled to protein A agarose beads (Pierce) in PBS containing 5 mg/mL of bovine serum albumin (Sigma-Aldrich) for 6 h at 4 °C on a rotating platform. The cell lysates were then incubated with the beads coupled with antibodies overnight at 4 °C. The next day, the beads were washed with lysis buffer containing an additional 300 mM of NaCl, and the immune complexes were eluted off the beads using loading buffer with boiling for 5 min and then subjected to Western blot analysis.

### 4.8. Microscopy and Imaging

Images were acquired using either an imaging microscope (Nikon TE 2000-U) with a Plan Apochromat 60X 1.49 oil objective (Nikon, Tokyo, Japan) or an Elyra PS.1 Super Resolution microscope equipped with a Plan Apochromat 63X 1.49 oil objective (Zeiss, White Plains, NY, USA). For ciliary length measurements, the region of interest was manually defined using the line segment tool. For ciliary intensity measurements of acetylated tubulin, the axoneme was segmented, and then a background subtraction was performed using the same-sized region of interest as the segmented cilium. The average pixel intensity was determined for the protein of interest using the Intensity module. Ciliary length and intensity measurements were performed using ImageJ.

### 4.9. Transmission Electron Microscopy (TEM)

mIMCD3 cells seeded on coverslips were grown to confluency and serum-starved. Cells were washed 1x with PBS, and pellets were re-suspended in Trump’s fixative (4% PFA and 1% glutaraldehyde). Cells were then post-fixed with 1% osmium tetroxide in cacodylate buffer and embedded in Embed812 resin according to standard procedures. Thin sections from the resin blocks were collected and mounted on a grid for viewing on an electron microscope (JEM-1400; JEOL, Inc., Peabody, MA, USA).

### 4.10. Real-Time Quantitative Reverse Transcription PCR (qRT-PCR)

Total RNA was extracted using the RNeasy Plus Mini Kit (QIAGEN, Germantown, MD, USA). Total RNA (1 μg) was used for RT reactions in a 20 μL reaction to synthesize cDNA using an iScript cDNA Synthesis Kit (Bio-Rad, Hercules, CA, USA). RNA expression profiles were analyzed by real-time PCR using iTaq SYBR Green Supermix with ROX (Bio-Rad) in an iCycler iQ Real-Time PCR Detection System. The complete reactions were subjected to the following program of thermal cycling: 40 cycles of 10 s at 95 °C and 20 s at 60 °C. A melting curve was run after the PCR cycles, followed by a cooling step. Each sample was run in triplicate in each experiment, and each experiment was repeated 3 times. The expression levels of target genes were normalized to the expression level of actin.

### 4.11. Chromatin Immunoprecipitation (ChIP) Assay

ChIP assays were performed according to the protocol described in the study [40]. Chromatin DNA was subjected to immunoprecipitation with an anti-SMYD3 antibody or normal rabbit IgG and then washed, after which the DNA–protein crosslinks were reversed. The recovered DNA was analyzed by PCR for the binding of SMYD3 at the mouse C2cd3, Cep164, Ttbk2, Dync2h1 and Cp110 promoters.

### 4.12. RNA Interference

The RNA oligonucleotides that specifically targeted mouse SMYD3 and mouse Ttbk2 were purchased from Santa Cruz Biotechnology Inc. The RNA oligonucleotides were transfected using Lipofectamine RNAiMAX (Invitrogen) following the manufacturer’s instructions. Forty-eight hours after transfection, the cells were harvested and analyzed by Western blotting.

### 4.13. Statistics

All data are presented as the mean ± SEM. All statistical analyses were performed using SPSS Statistics 22 software. *p* values were calculated by a 2-tailed unpaired Student’s t-test and a 1-way ANOVA, and a *p* value less than 0.05 was considered significant.

## Data Availability

The authors declare that the data supporting the findings of this study are available within the article and the Supplementary Materials, or from the corresponding author upon reasonable request.

## Supplementary Materials

The following supporting information can be downloaded at: https://www.mdpi.com/article/10.3390/ijms25116040/s1.
