# Supplementary material for: SMYD3 Controls Ciliogenesis by Regulating Distinct Centrosomal Proteins and Intraflagellar Transport Trafficking
Source: Int J Mol Sci. 2024 May 30;25(11):6040. doi: 10.3390/ijms25116040 (PMC11172885; doi:10.3390/ijms25116040)
Supplement: Supplementary file 1 [file ijms-25-06040-s001.zip › ijms-2981427-supplementary.pdf]

Supplementary Figure S1

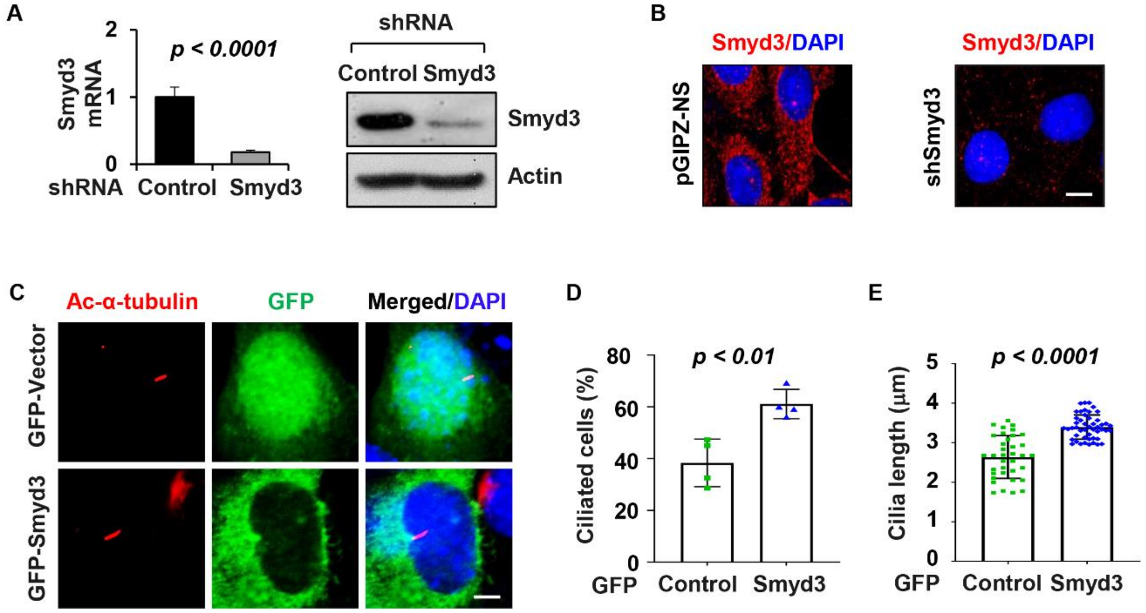

Supplemental Figure S1. Exogenously expressed GFP-tagged SMYD3 enhances ciliogenesis. (**A** and **B**) Mouse IMCD3 cells were transduced with Lentivector-mediated SMYD3 shRNA, pGIPZ–shSMYD3, and the control vector, pGIPZ-NS, respectively. The knockdown efficiency of SMYD3 in these cells was analyzed by qRT-PCR and western blot (**A**), and by immunostaining (**B**) with SMYD3 antibody (red) and DAPI (blue). Scale bars, 10 µm. (**C** – **E**) Overexpression of SMYD3 enhances ciliogenesis. Representative images (**C**), and quantification analysis of GFP positive cells (n>150) with cilia (**D**), and average cilia length (**E**) (n>45), in GFP-SMYD3 overexpressed NIH3T3 cells compared to GFP-vector control cells. Scale bar, 5 µm. Error bars represent the SD.

Supplementary Figure S2

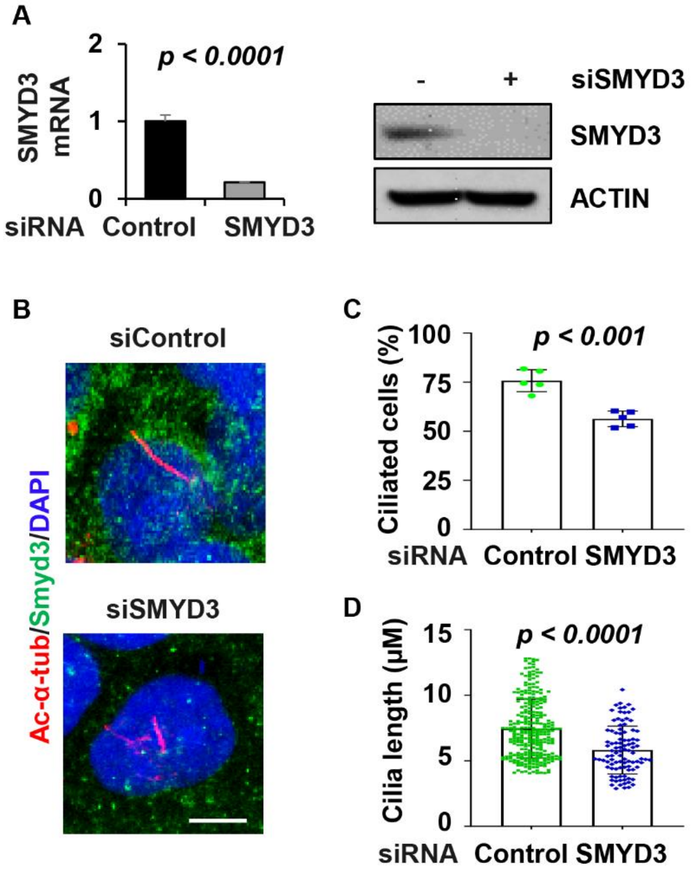

Supplemental Figure S2. Knockdown of SMYD**3 inhibits ciliogenesis in RCTE cells.**

RCTE cells were transduced with SMYD3 siRNA and control siRNA. (**A**) The knockdown efficiency of SMYD3 in RCTE cells analyzed by qRT-PCR and western blot. (**B** – **D**) Knockdown of SMYD3 inhibits ciliogenesis. Representative image (**B**), and quantification of the percentage of ciliated cells (n>200) (**C**), and average cilia length (n>100) (**D**), in SMYD3 knockdown RCTE cells compared to those in siRNA control RCTE cells. Scale bars, 20 μm.

Supplementary Figure S3

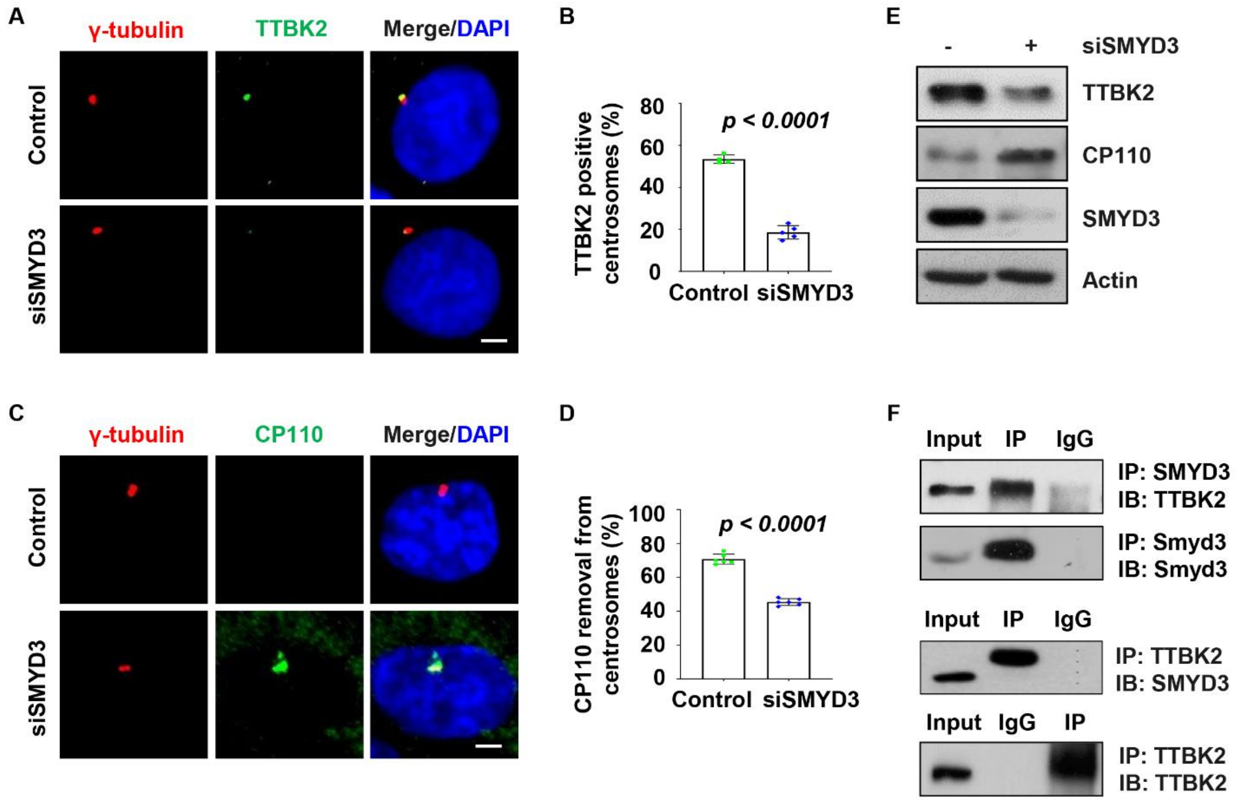

Supplemental Figure S3. SMYD3 forms a complex with TTBK2. (**A** and **B**) Representative images (**A**), and quantitative analysis of TTBK2 centriole localization (**B**) in SMYD3 knockdown RCTE cells stained with TTBK2 and γ-tubulin. Analysis indicated that TTBK2 was removed from the centriole in siSMYD3 knockdown RCTE cells but not in siRNA control RCTE cells. Scale bars, 5 μm. (**C** and **D**) Representative images (**C**), and quantitative analysis of CP110 centriole localization (**D**) in SMYD3 knockdown RCTE cells stained with CP110 and γ-tubulin. Analysis indicated that CP110 was removed from the centriole in siRNA control RCTE cells but not in siSMYD3 knockdown RCTE cells. Scale bars, 5 μm. (**E**) Western blot analysis of TTBK2 and CP110 protein levels in SMYD3 knockdown and siRNA control RCTE cells. (**F**) SMYD3 forms a complex with TTBK2 in RCTE cells as examined by co-immunoprecipitation analysis. 5% input was used.

Supplementary Figure S4

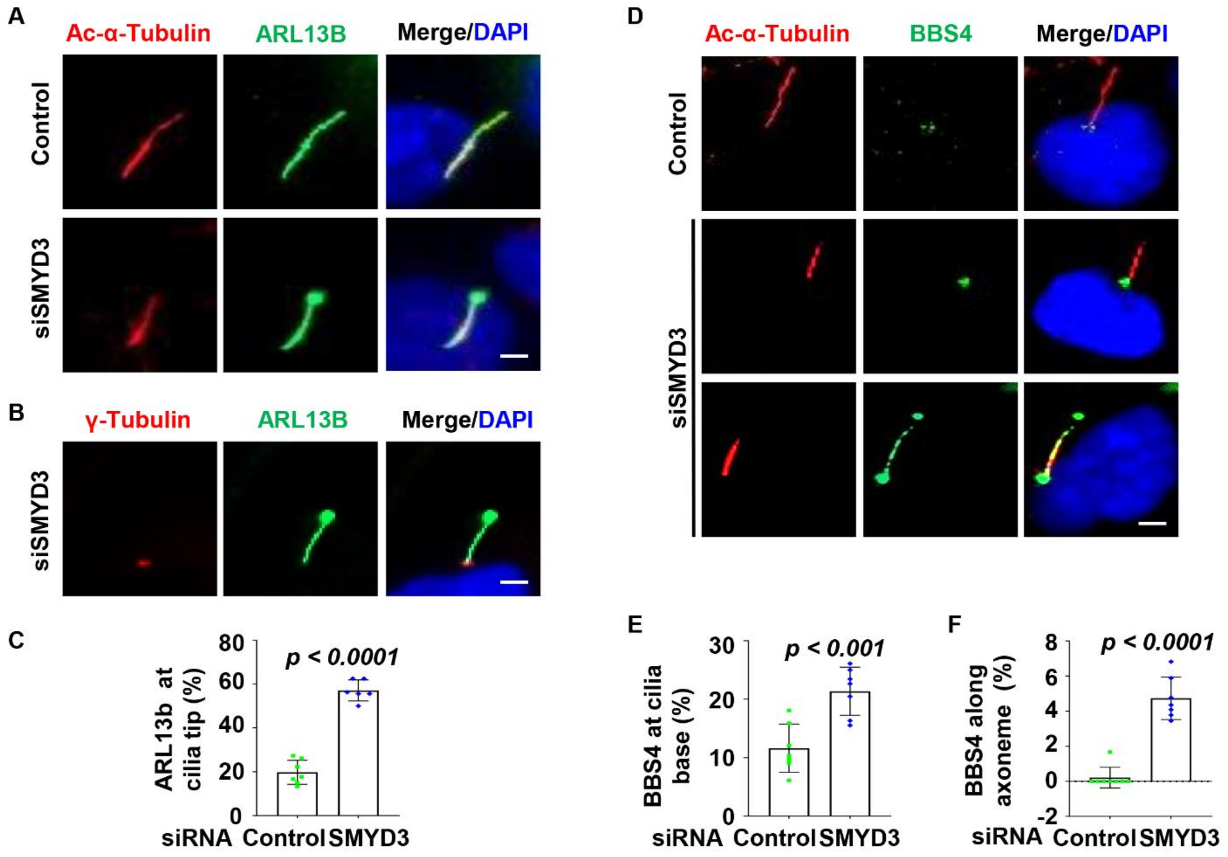

Supplemental Figure S4. Knockdown of SMYD3 increases the ciliary accumulation of ARL13B and BBS4. (A and B) Representative images of ARL13B (green) co-stained with acetylated-α-tubulin (red) (A), and ARL13B (green) co-stained with γ-tubulin (red) (B), in siSMYD3 knockdown RCTE cells. (C) Quantitative analysis of the percentage of ciliated cells with ciliary tip accumulation of ARL13B (n>150), in SMYD3 knockdown RCTE cells. Scale bars, 5 μm. (D) Representative images of BBS4 (green) co-stained with acetylated-α-tubulin (red) in siSMYD3 knockdown RCTE cells. (E and F) Quantitative analysis of the percentage of ciliated cells with BBS4 cilia base (n>80) (E), and axoneme accumulation (n>100) (F) in SMYD3 knockdown RCTE cells. Scale bars, 5 μm.

Supplementary Figure S5

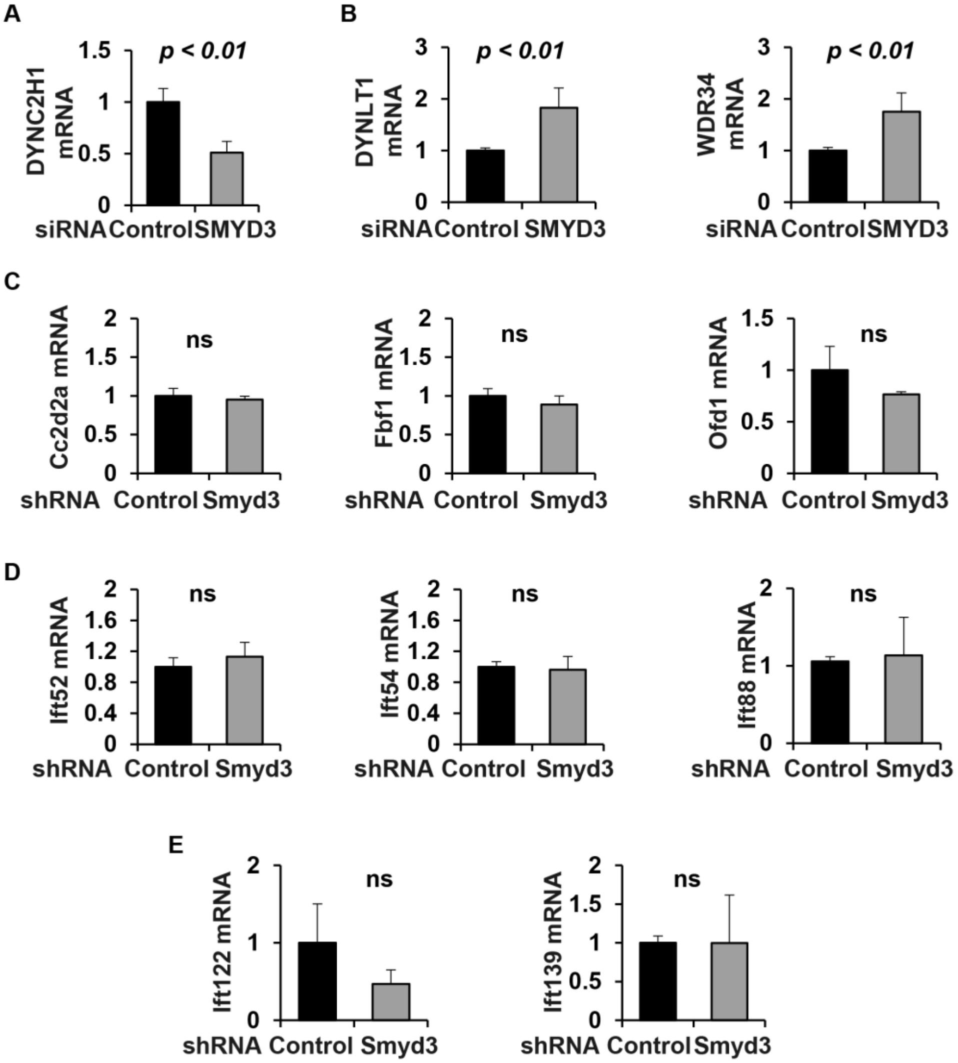

Supplemental Figure S5. SMYD3 regulates the transcription of cilia related genes. (**A** - **E**) qRT-PCR analysis of mRNA levels of key ciliary genes. qRT-PCR analysis reveals a decrease in the mRNA level of dynein heavy chain component DYNC2H1 (**A**), and a decrease in the mRNA levels of dynein light chain DYNLT1, and dynein intermediate chain WDR34, components (**B**), in SMYD3 siRNA depleted RCTE cells compared to controls. qRT-PCR analysis reveals no change in the mRNA levels of the centriole appendage components (Cc2d2a, Fbf1, and Ofd1) (**C**), anterograde transport components (Ift52, Ift54, and Ift88) (**D**), and retrograde transport components (Ift122 and Ift139) (**E**), in SMYD3 depleted IMCD3 cells compared to control cells.
